# Prions in Variably Protease-Sensitive Prionopathy: An Update

**DOI:** 10.3390/pathogens2030457

**Published:** 2013-07-05

**Authors:** Wen-Quan Zou, Pierluigi Gambetti, Xiangzhu Xiao, Jue Yuan, Jan Langeveld, Laura Pirisinu

**Affiliations:** 1 Department of Pathology Case Western Reserve University School of Medicine, Cleveland, OH 44106, USA; E-Mails: pxg13@case.edu (P.G.); xiangzhu.xiao@case.edu (X.X.); jue.yuan@case.edu (J.Y.); 2 Department of Neurology, Case Western Reserve University School of Medicine, Cleveland, OH 44106, USA; 3 National Prion Disease Pathology Surveillance Center, Case Western Reserve University School of Medicine, Cleveland, OH 44106, USA; 4 National Center for Regenerative Medicine, Case Western Reserve University School of Medicine, Cleveland, OH 44106, USA; 5 The First Affiliated Hospital, Nanchang University, Nanchang 330006, Jiangxi Province, China; 6 State Key Laboratory for Infectious Disease Prevention and Control, National Institute for Viral Disease Control and Prevention, Chinese Center for Disease Control and Prevention, Beijing 100050, China; 7 Central Veterinary Institute of Wageningen UR, Lelystad 8200 AB, the Netherlands; E-Mail: jan.langeveld@wur.nl (J.L.); 8 Department of Veterinary Public Health and Food Safety, Istituto Superiore di Sanità, Viale Regina Elena 299 00161, Rome, Italy; E-Mail: laura.pirisinu@guest.iss.it (L.P.)

**Keywords:** : Prions, prion protein, prion disease, Creutzfeldt-Jakob disease (CJD), variably protease-sensitive prionopathy (VPSPr), Gerstmann-Sträussler-Scheinker (GSS), mutation, proteinase K, antibody, glycosylation, glycoform-selective prion formation, transmissibility

## Abstract

Human prion diseases, including sporadic, familial, and acquired forms such as Creutzfeldt-Jakob disease (CJD), are caused by prions in which an abnormal prion protein (PrP^Sc^) derived from its normal cellular isoform (PrP^C^) is the only known component. The recently-identified variably protease-sensitive prionopathy (VPSPr) is characterized not only by an atypical clinical phenotype and neuropathology but also by the deposition in the brain of a peculiar PrP^Sc^. Like other forms of human prion disease, the pathogenesis of VPSPr also currently remains unclear. However, the findings of the peculiar features of prions from VPSPr and of the possible association of VPSPr with a known genetic prion disease linked with a valine to isoleucine mutation at residue 180 of PrP reported recently, may be of great importance in enhancing our understanding of not only this atypical human prion disease in particular, but also other prion diseases in general. In this review, we highlight the physicochemical and biological properties of prions from VPSPr and discuss the pathogenesis of VPSPr including the origin and formation of the peculiar prions.

## 1. Introduction

Prions are infectious pathogens that are associated with a group of fatal transmissible spongiform encephalopathies or prion diseases affecting both animals and humans. They are composed mainly, if not entirely, of the pathologic scrapie conformer (PrP^Sc^) and originate from the cellular prion protein (PrP^C^) by means of a structural transition from a largely α-helical form to predominantly β-sheets [1]. Unlike other infectious agents, such as bacteria, viruses, and fungi, which contain genomes composed of either DNA or RNA, prions are the only known infectious pathogens that are devoid of nucleic acid, according to the “protein only” hypothesis [1]. Human prion diseases are highly heterogeneous: They can be familial, sporadic, or acquired by infection, and include Creutzfeldt-Jakob disease (CJD), Gerstmann-Sträussler-Scheinker (GSS) disease, fatal insomnia, kuru and variant CJD (vCJD) [2]. Atypical human and animal prion diseases have recently been identified including variably protease-sensitive prionopathy (VPSPr) in human and Nor98/atypical scrapie in sheep and goats [3,4,5,6,7]. The two atypical human and sheep prion diseases are characterized by the deposition of peculiar prions in the brain. No mutations have been found in the open reading frame of prion protein gene in the two diseases. While Nor98 scrapie is associated with polymorphisms at R154H and L141F, VPSPr is observed in all three genotypes of PrP polymorphism at residue 129 of PrP. PrP^Sc^ from the two diseases exhibited a small PK-resistant fragment similar to those observed in some of familial prion diseases [4,5,6,8].

## 2. Dominant Protease-Sensitive PrP^Sc^ Conformer

In the eleven cases first reported, they were all valine/valine homozygosity at residue 129 of PrP and more than half of them had a family history of dementia [4]. Although spongiform degeneration and PrP immunostaining were observed, surprisingly, no typical PK-resistant PrP^Sc^ (rPrP^Sc^) was detectable in the brain of all cases by conventional Western blotting probing with the widely-used anti-PrP antibody 3F4. The 3F4 antibody that has an epitope between residues 106 and 112 [9] detected an abnormal PrP in PSPr only after enrichment with gene 5 protein (g5p) and sodium phosphotungstate (NaPTA) that are able to bind to abnormally-folded PrP molecules regardless of their PK resistance [10,11]. However, more than 70% of the abnormal PrP captured by g5p from these cases was sensitive to PK-digestion while only about 10% of captured PrP^Sc^ was PK-sensitive in sCJD. Therefore, this atypical human prion disease characterized by the deposition in the brain of dominant PK-sensitive PrP^Sc^ (sPrP^Sc^) was initially termed as protease-sensitive prionopathy (PSPr) [4].

**Figure 1:**
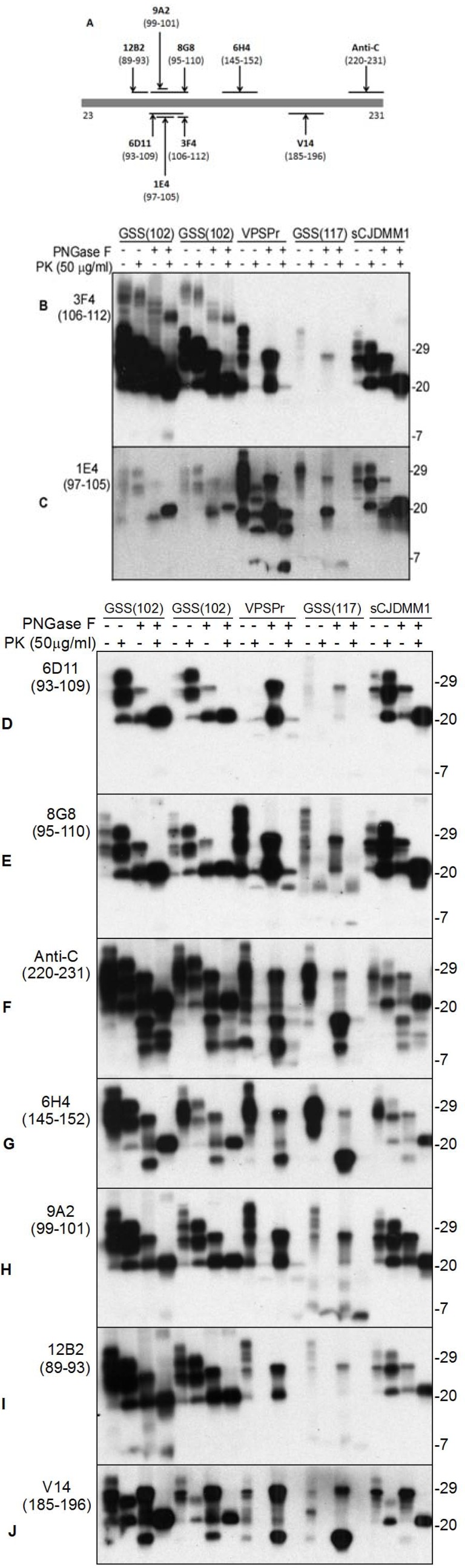
Detection of PrP from variably protease-sensitive prionopathy (VPSPr), Gerstmann-Sträussler-Scheinker (GSS), and sporadic Creutzfeldt-Jakob disease (sCJD) with 129 methionine/methionine (MM) polymorphism and PrP^Sc^ type 1 (sCJDMM1) with nine anti-PrP antibodies. ***A*:** Diagram of epitope locations of anti-PrP antibodies examined on human PrP. Antibodies and their epitopes are: 3F4 (PrP106-112), 1E4 (PrP97-105), 6D11 (PrP93-109), 8G8 (PrP95-110), Anti-C (PrP220-231), 6H4 (PrP145-152), 9A2 (PrP99-101), 12B2 (PrP89-93), and V14 (PrP185-196). ***B*** through ***J:*** Brain homogenates from VPSPr, GSS linked to PrP^P1^°^2L^ mutation (GSS102), GSS linked to PrP^A117V^ mutation, and sCJDMM1 were treated with PK or/PNGase F prior to SDS-PAGE and Western blotting with nine different anti-PrP antibodies, respectively. ***B:*** 3F4; ***C:*** 1E4; ***D:*** 6D11; ***E:*** 8G8; ***F:*** Anti-C; ***G:*** 6H4; ***H:*** 9A2; ***I:*** 12B2; ***J:*** V14. Of the nine antibodies used, 1E4 exhibits the highest affinity for rPrP^Sc^ from VPSPr. However, 1E4 has a lower affinity for rPrP^Sc^ from GSS102 compared to 3F4. It could be due to the PrP^P102L^ mutation that is localized within the 1E4 epitope. Since VPSPr20 and VPSPr17 are detectable by 6D11 that is against human PrP93-109, their N-terminal domains may start at least from residue 93. VPSPr7 is recognized by 1E4 that is against human PrP97-105, suggesting that the N-terminus of VPSPr7 contains residue 97.

## 3. Pathognomonic Ladder-Like Electrophoretic Profile of Protease-Resistant PrP^Sc^ with a Peculiar Immunoreactivity Behavior

Although undetectable with 3F4 by conventional Western blotting, a ladder-like electrophoretic profile of PK-resistant PrP^Sc^ (rPrP^Sc^) was readily detected with an antibody called 1E4, which is unprecedented in sporadic human prion diseases [4]. The 1E4 clone was derived from hybridization of SP2/0-Ag14 myeloma cells with spleen cells from a PrP-knockout mouse immunized with the peptide QWNKPSKPKTN that corresponds to the bovine PrP amino acid sequence 108–119 (http://www.cellsciences.com/content/p-detail.asp?rowid=8107). While this antibody was mostly used for detection of bovine and ovine PrP^Sc^, we demonstrated that this antibody with an epitope localized between human PrP residues 97 and 105 (Yuan *et al.*, 2008) exhibiting the highest affinity for PrP^Sc^ of PSPr among the nine anti-PrP antibodies we examined including 1E4 (against PrP97-105), 3F4 (PrP106-112), 6D11 (PrP93-109), 8G8 (PrP95-110), Anti-C (220-231), 6H4 (PrP145-152), 9A2 (PrP99-101), 12B2 (PrP89-93), and V14 (PrP185-196) (Figure 1). Since the 1E4-detected ladder-like electrophoretic profile of rPrP^Sc^ is very unique and was detected repeatedly in all eleven cases, it has been considered to be pathognomonic for PSPr. 

The discovery of the pathognomonic molecular feature of PSPr has greatly facilitated the identification of this unique type of human prion disease. Bearing this feature of PSPr in mind, we reexamined retroprospectively suspected cases referred to the National Prion Disease Pathology Surveillance Center (NPDPSC, Cleveland, OH, USA) between 2002 and 2010 including two cases from Italy [5]. For newly-referred cases, it has become a routine procedure to re-do Western blot analysis with the 1E4 antibody in order to find out the possible cases of PSPr at NPDPSC if they are negative for rPrP^Sc^ by Western blotting with 3F4 but positive for H&E staining and immunohistochemistry with 3F4. 

## 4. Polymorphism-Dependent PK-Sensitive and PK-Resistant PrP^Sc^

In 2010, we first reported that PSPr affects not only subjects homozygous for valine at PrP residue 129 but also subjects homozygous for methionine (129 MM) or heterozygous for methionine/valine (129 MV) [5]. Of the fifteen cases we examined, one of the two Italian cases was previously reported by Giaccone et al [3]. Compared to the initially reported PSPr in valine homozygotes, the levels of sPrP^Sc^ were significantly decreased while the levels of rPrP^Sc^ were significantly increased in 129 MM or 129 MV cases. Interestingly, it seems that the levels of rPrP^Sc^ are dictated by methionine at residue 129. Vice versa, the levels of sPrP^Sc^ seem to be dictated by valine at residue 129. Although it has been well-documented that PrP polymorphism at residue 129 is implicated in mediating susceptibility to the disease, phenotypes of disease, and PrP^Sc^ types [2], to our knowledge, our study provided the first evidence that the polymorphism may also participate in medicating the amounts of sPrP^Sc^ or rPrP^Sc^ [5]. To more precisely reflect the polymorphism-dependent variation in the levels of rPrP^Sc^ or sPrP^Sc^ in this newly-identified disease, we revised the original designation as “variably protease-sensitive prionopathy” (VPSPr) [5]. 

**Figure 2:**
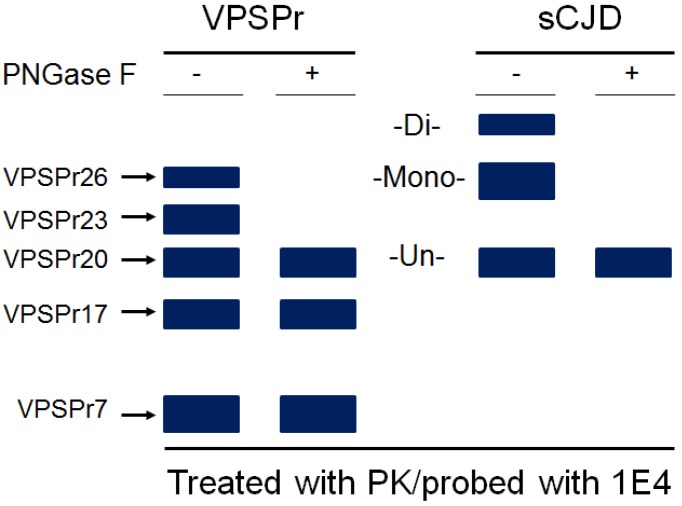
**Schematic diagram of electrophoretic profile of rPrP^Sc^ from VPSPr and sCJD probed with 1E4.** Without PNGase F treatment, five rPrP^Sc^ fragments are detectable with Western blotting including VPSPr26, VPSPr23, VPSPr20, VPSPr17, and VPSPr7 from VPSPr while three rPrP^Sc^ fragments are detected including di-, mono-, and un-glycosylated PrP from classic sCJD. After PNGase F treatment, three core PrP fragments remain in VPSPr including VPSPr20, VPSPr17, and VPSPr7 while only one core PrP fragment remains in sCJD. VPSPr26 and VPSPr23 are monoglycosylated forms of VPSPr20 and VPSPr17, respectively.

## 5. The Ladder-Like Electrophoretic Profile of 1E4-Detected rPrP^Sc^ Consisting of Five PrP Fragments

Some of the rPrP^Sc^ fragments became detectable in VPSPr129MM and VPSPr129MV with the 3F4 antibody, especially in the former. Notably, even though the amounts of rPrP^Sc^ in VPSPr129MM or VPSPr129MV were significantly increased compared to those of rPrP^Sc^ in VPSPr129VV, the profile of rPrP^Sc^ detected with 3F4 is different from that detected with 1E4. The most significant differences in the rPrP^Sc^ fragments detected with the two antibodies were the smallest fragment migrating at approximately 7 kDa called VPSPr7 that was detectable with 1E4 but not with 3F4 [5,6]. Except for VPSPr7, both 3F4 and 1E4 detected the other four rPrP^Sc^ fragments migrating at ~26 kDa, 23 kDa, 20 kDa, and 17 kDa, termed VPSPr26, VPSPr23, VPSPr20, and VPSPr 17, respectively, which is strikingly different from rPrP^Sc^ observed in classic sCJD (Figure 2). Based on gel migration, VPSPr26 corresponds to monoglycosylated rPrP^Sc^ of the classic sCJD, whereas VPSPr20 corresponds to unglycosylated rPrP^Sc^ of sCJD. Interestingly, no detectable diglycosylated rPrP^Sc^ was observed by both 3F4 and 1E4 in VPSPr, which was also observed in the first case reported [3]. On the other hand, three additional fragments VPSPr23, VPSPr17 and VPSPr7 were not detectable in sCJD. A PK-titration of PrP^Sc^ from VPSPr129MM or 129MV on the Western blots probed with 1E4 or 3F4 revealed that VPSPr26 and VPSPr20 gradually faded away while VPSPr23 and VPSPr17 became dominant upon increases in the PK concentrations, suggesting that VPSPr23 derives from VPSPr26 while VPSPr17 from VPSPr20. After deglycosylation, we convincingly showed that unglycosylated VPSPr20 decreased while VPSPr17 increased. As mentioned above, VPSPr20 may correspond to unglycosylated rPrP^Sc^ of sCJD and it may encompass PrP sequence from residue 90 to 231. So, it is most likely that the switch from VPSPr20 to VPSPr17 caused by PK-digestion may be due to cleavage of a C-terminal domain since both fragments were detected with antibodies against human PrP97-105 (1E4) and PrP106-112 (3F4). Moreover, the antibody Anti-C specific for human PrP220-231 detected four rPrP^Sc^ after deglycosylation with PNGase F. However, the migration of these fragments detected with Anti-C seemed to be different from that of 1E4-detected fragments except for VPSPr20. All these findings suggest that conformation of PrP^Sc^ in VPSPr is quite different from that of PrP^Sc^ in sCJD and it may have many more PK-cleavage sites of PrP^Sc^ in VPSPr than in sCJD. The cleavages may occur not only at the N-terminal domain but also at the C-terminal domain.

## 6. Glycoform-Selective Prion Formation in VPSPr and fCJD^V180I^

In general, the four glycoforms of PrP^C^ variably glycosylated at the two N-linked glycosylation sites are usually all converted into their PrP^Sc^ counterparts in all human prion diseases [2]. However, there are exceptions. Familial CJD linked to either PrP^T183A^ (fCJD^T183A^) or PrP^V180I ^(fCJD^V180I^) mutation exhibits an rPrP^Sc^ that lacks the diglycosylated PrP species [12,13]. T183A mutation itself is known to completely eliminate the first glycosylation site at residue 181 [14], which is believed to account for the lack of diglycosylated rPrP^Sc^. However, the molecular mechanism underlying alteration in glycosylation by V180I mutation is unknown. In addition to the two mutations, two cases of atypical sporadic CJD have also been reported to lack diglycosylated rPrP^Sc^ [3,15], one of which was subsequently proven to be a case of VPSPr [3,5]. 

To understand the molecular mechanism underlying the lack of diglycosylated rPrP^Sc^ and formation of ladder-like electrophoretic profile of rPrP^Sc^ as well as to investigate the potential association between sporadic VPSPr and familial CJD lacking diglycosylated PK-resistant PrP form, we compared their individual PrP glycoforms using antibodies that are able to distinguish the two glycosylation sites [6,16,17]. Using a combination of *in vivo* and *in vitro* assays, we demonstrated that the absence of the diglycosylated PrP^Sc^ in both VPSPr and fCJD^V180I^ is associated with the inability of the di- and mono-glycosylated PrP^C^ with the intact first glycosylation site (N181) to convert into PrP^Sc^ in the brain. Surprisingly, fCJD^V180I^ was detected to have an rPrP^Sc^ that was markedly similar to that observed in VPSPr, with a five-step ladder-like electrophoretic profile, a pathognomonic molecular feature of VPSPr [6]. Therefore, although VPSPr is linked to wild-type PrP and fCJD^V180I^ is linked to mutant PrP, both sporadic VPSPr and fCJD^V180I^ may share a unique glycoform-selective prion formation pathway. Moreover, conformation of PrP^Sc^ in the two conditions also may be very similar, as evidenced by the generation of a virtually identical electrophoretic profile of rPrP^Sc^ upon PK-treatment.

Although the molecular mechanism underlying glycoform-selective prion formation is unclear at present, there are several clues that may be of significance in understanding this mystery. First, in contrast to fCJD^T183A^, both VPSPr and fCJD^V180I^ exhibited an intact glycosylation prior to PK-digestion. Moreover, PrP^V180I^ in cultured cells had a typical glycoform profile. It generated detectable typical rPrP^Sc^ with diglycosylated PrP form upon PK-treatment, in addition to mono-unglycosylated forms although they were detectable only with 1E4 but not with 3F4 [6]. Therefore, PrP^V180I^ mutation itself does not eliminate any glycosylation sites and it can be converted into rPrP^Sc^ as does wild-type PrP^C^. Second, a decrease in the glycosylation potential value for the first glycosylation site was predicated by the N-linked glycosylation prediction algorithm [6], suggesting that PrP^V180I^ may alter the composition of glycans at the first site. Third, diglycosylated PrP or monoglycosylated PrP carrying mono181 was not converted into rPrP^Sc^, which was not observed in cultured cells but only observed in the brain in which there is an additional wild-type allele. Finally, compared to sCJD, the binding of *ricinus communis agglutinin* I (RCA-I) to monoglycosylated PrP decreased while the binding to diglycosylated PrP increased in VPSPr and fCJD^V180I^ [6], suggesting that the two diseases have a changed composition of glycans. Therefore, it is possible that glycoform-selective prion formation observed in brain involves dominant-negative inhibition caused by the interaction between misfolded and normal PrP molecules. The changed composition of glycans at the first site by the mutation may alter local conformation around residue 181 that is close to the β-sheets 2/α-helixes 2 loop, the critical region implicated in dominant-negative inhibition [18]. Consistent with this hypothesis, it seems that there are significant differences in the effect of mutations occurring at either the first or the second glycosylation site on the conversion of PrP^C^ into PrP^Sc^. While most of mutations at the first site blocked the conversion, none of or a few, if any, mutations at the second site were found to block PrP^C^ conversion in cell and animal models [19,20]. More specifically, interactions between different PrP^C^ glycoforms mediate the efficiency of prion formation, which involves glycan-associated steric hindrance [21]. The same group also demonstrated that dominant negative inhibition of prion formation requires no protein X or any other accessory cofactor [22]. Although no PrP mutations have been observed in VPSPr, a similar aberrant glycosylation at N181 caused by a rare stochastic event has been proposed to trigger the processes as described for fCJD^V180I^ [6]. 

Another possibility to cause glycoform-selective prion formation is that one or more co-factors may be operating in VPSPr and fCJD^V180I^ and the co-factors may prevent conversion of diglycosylated PrP and mono181 into PrP^Sc^. There may be some implications in the two diseases that other co-factors may be involved in the pathogenesis of the diseases. For instance, although linked to the PrP^V180I^ mutation, no family history of neurodegenerative disorders has been reported in fCJD^V180I^ cases [23]. On the other hand, while no mutations have been identified within the coding sequence (the open reading frame) of PrP gene in VPSPr to date, eight out of 26 reported VPSPr cases showed a familial history of dementia [5,24]. Indeed, several lines of evidence have indicated that other co-factors may be involved in the pathogenesis of prion diseases, including protein X and non-proteinaceous cofactors [25,26,27]. Whether protein X that was initially proposed to directly interact with PrP^C^ is necessary for prion formation remains controversial [26,28]. However, genes or proteins that may indirectly trigger the conversion of PrP^C^ into PrP^Sc^ may exist. It is possible that a mutation in a specific gene that participates in regulating PrP glycosylation may alter glycosylation at the first glycosylation site and then causes VPSPr or/and fCJD^V180I^. If this is the case, further investigation of the two diseases may provide an opportunity to find out about the existence of such a co-factor. 

## 7. Transmissibility of VPSPr and fCJD^V180I^

It has been widely accepted that any prion diseases must be transmissible although there are some prion diseases that have not been transmitted yet [29,30]. Before Stanley Prusiner discovered prions and coined *prion* in 1982 [31], the term *transmissible* *spongiform* *encephalopathy* (TSE) had been widely used. As indicated by its striking name, a TSE must possess these two major characteristics: transmissibility and spongiform degeneration in the central nervous system (CNS). The discovery of prions as infectious protein pathogens, which are free of nuclear acids and which are the cause of transmissible spongiform encephalopathies, was a revolutionary development not only for the field in particular but also for the life science in general. PrP^Sc^ has in fact been observed in almost all TSE identified so far. Identification of PrP^Sc^ has become essential in the current diagnostic criteria for TSE. The designation, “prion diseases”, has largely replaced “transmissible spongiform encephalopathy”.

However, confusion results when it is observed that some prion diseases lack one or two characteristics of TSE. Approximately 10% of sporadic CJD and 32% of familial prion diseases were non-transmissible in nonhuman primates [32]. Moreover, all GSS, except one-third of GSS^P102L^ cases, were difficult to transmit to rodents [33]. Further, the spongiform degeneration typical of TSE is not always present in all GSS^P102L^, although diffuse deposits of PrP^Sc^ plus PrP-amyloid plaques are present in the CNS [34]. In transgenic mice expressing murine PrP P101L (equivalent to human P102L) and challenged with GSS free of spongiform degeneration, neither symptoms nor spongiform degeneration was observed despite the presence of PrP-amyloid [35]. Obviously, conditions such as these, which do not manifest transmissibility or spongiform degeneration (singly or jointly), should not be considered as types of TSE. They do, however, constitute prion diseases. Based on a wealth of data gathered so far, one may wonder whether or not prion diseases should now be redefined. Under a reconsidered definition, they should include a group of disorders characterized by the accumulation of abnormal PrP including protease-sensitive and protease-resistant forms in the brain, regardless of the presence of transmissibility and spongiform degeneration. Most importantly, the spectrum of prion diseases must not be restrained by the definition of TSE.

As a prion disease, VPSPr should likewise meet Koch’s postulates as well [36]. So, it would be important to investigate the transmissibility of VPSPr that exhibits the peculiar PK-resistant behavior of PrP. Preliminary data recently reported by Nonno et al. indicate that transmissibility of VPSPr-129MM, -129MV and -129VV to bank voles is related to the bank vole methionine / isoleucine polymorphism at codon 109 [37]. Furthermore, studies with humanized mice suggest that transmissibility of VPSPr is much lower compared to classic sporadic CJD [38]. No clinical phenotypes were observed during the normal life span of transgenic mice expressing human PrP-129V at approximately 10 fold, following inoculation with brain homogenates from VPSPr-129VV cases. Less than 20% of the mice were found to have scattered PrP plaques with minimal or no spongiform degeneration, compared to the typical neuropathological changes found in 100% mice inoculated with the classic sCJD [38]. Similarly, using protein misfolding cyclic amplification (PMCA) assay, we found that the amplification efficiency of PrP^Sc^ from VPSPr is much lower compared to iatrogenic and sporadic CJD (Zou *et al*., unpublished data). To date, there are no reports available demonstrating that fCJD^V180I^ is transmissible [39]. Therefore, prions in VPSPr and fCJD^V180I^ exhibit striking similarities not only in physicochemical but also in biological properties. In collaboration with Drs. Yong-Sun Kim and Robert Petersen, we are generating humanized transgenic mice expressing human PrP^V180I^ currently and will be testing transmissibility of VPSPr and fCJD^V180I^ with this animal model.

## 8. Origin of Prions in VPSPr and fCJD^V180I^

As mentioned above, prions from VPSPr and fCJD^V18^°^I^ are of unique physicochemical and biological properties. Remarkably, they exhibit a high immunoreactivity with the 1E4 antibody but a poor reactivity with 3F4 [5,6]. We have demonstrated that the two antibodies have adjacent epitopes and especially the 3F4 epitope (PrP106-112) is next to the C-terminus of the 1E4 epitope (PrP97-105) [9,40]. Because of the unique localization of the two epitopes, it is most likely that all five-step like rPrP^Sc^ fragments from the two diseases contain the 3F4 epitope. So, the poor affinity of 3F4 for rPrP^Sc^ from VPSPr and fCJD^V180I^ may indicate that there might be some local structures or binding molecules that block the 3F4 epitope. We have noticed that the affinity of 3F4 for rPrP^Sc^ from VPSPr was increased in the preparations after purification steps compared to unpurified total brain homogenates (Zou *et al.*, unpublished data). Thus, purification procedures may somehow remove the binding molecules or alter the local structures, which might make the 3F4 epitope exposed. On the other hand, all these findings may also suggest that PrP^Sc^ from VPSPr and fCJD^V180I^ have an origin different from PrP^Sc^ detected in other human prion diseases. Using the same 1E4 antibody, we previously identified a PK-resistant PrP species termed insoluble PrP^C^ (iPrP^C^) in uninfected human brains and cultured cells [6,9,40,41,42]. The small amount of PK-resistant PrP in uninfected brains and cells exhibited the same peculiar immunoreactivity behavior: higher affinity for 1E4 but lower affinity for 3F4. Remarkably, the resemblances of three PK-resistant PrP core fragments migrating at ~20 kDa, 17 kDa and 7 kDa observed in VPSPr were detected with 1E4 in uninfected human brains [43]. The same immunoreactivity behavior of iPrP^C^ in uninfected brains and rPrP^Sc^ in VPSPr and fCJD^V180I^ suggests that they may share a common molecular metabolic pathway or distribution and that VPSPr and fCJD^V180I^ may result from an increase in the amount of iPrP^C^ [43].

## 9. Association between VPSPr and Other Prion Diseases

In addition to V180I and T183A mutations, three other naturally occurring PrP mutations including D178N, F198S, and E200K linked to familial prion disease have reportedly been associated with altered ratios of the three PrP glycoforms. But all the three familial prion diseases do not have rPrP^Sc^ that lacks diglycosylated form [39]. Moreover, the 1E4-preferentially detectable rPrP^Sc^ has not been identified yet. The deposition in the brain of multiple small PK-resistant PrP^Sc^, especially the 7-kDa fragment is the molecular hallmark of GSS [44]. Therefore, it is reasonable for us to anticipate some potential association between GSS and VPSPr. Indeed, because of the long disease duration, multiple PK-resistant PrP fragments, and variable PK-resistance of PrP^Sc^, VPSPr was once suspected to be the sporadic form of GSS associated with PrP^A117V^ mutation (GSS^A117^) [5]. However, we also observed different ratios and immunoreactivity of PrP^Sc^ between VPSPr and GSS^A117V^ in the same study. It is known that GSS is frequently associated with a predominantly cerebellar dysfunction and is mainly characterized by the deposition of multicentric plaques in the cerebellum [39]. In contrast, VPSPr lacks typical multicentric plaques while it exhibits dot-like staining or small plaque-like formations in the cerebellum [5]. Whether VPSPr is the sporadic form of fCJD^V180I^ or GSS^A117V^ needs to be further determined. It is conceivable that cells and animals expressing human PrP^V180I^ or PrP^A117V^ will provide valid models for addressing the outstanding questions.

The fact that a small PK-resistant rPrP^Sc^ migrating at ~6–7 kDa that has been believed to be a molecular hallmark of various GSS is also detectable in both VPSPr and Nor98 [2,5,7,34,35,44] may imply a possible association among these diseases. To gain insights into their apparent similarity and difference and to investigate possible relationships among them, we further compared the small fragment from VPSPr, Nor98, various GSS linked to P102L, A117V, or F198S PrP mutation [8,45]. It was demonstrated that VPSPr and Nor98 share both similar and distinctive features. For instance, interestingly, they all have a core rPrP^Sc^ fragment encompassing PrP97-142 while the fragment can have varied N- and C-terminal cleavage sites (Table 1) [45]. 

**Table 1:** Antibody mapping of the 6–7 kDa small rPrP^Sc^ [45].

MAbs	Epitopes	Human					Sheep
		VPSPr	A117	F198S	102	sCJD	Nor98
SAF32	Octarepeat	–	–	+	+	+	–
12B2	89–93	–	+	+	+	+	+
9A2	99–101	±	+	+	+	+	+
6D11	93–109	+	+	+	+	+	+
8G8	95–110	+	+	+	+	+	+
F89	139–142	+	+	+	+	+	+
L42	145–150	+	±	±	+	+	+
12F10	143–152	+	–	–	–	+	–

Repeat region amino acids: 59–65; 67–73; 75–81; 83–89–: No signal; +: strong signal; and +/–: week signal.

## 10. Conclusions

Prions found in sporadic VPSPr are clearly different from those of all other classic sporadic human prion diseases. Both VPSPr and fCJD^V180I^ shares similar physicochemical properties of PrP^Sc^ and a glycoform-selective prion formation pathway. The finding of the effect of polymorphism at residue 129 on the levels of rPrP^Sc^ and sPrP^Sc^ further emphasizes the role of the polymorphism in the pathogenesis of human prion diseases. The two diseases specifically alter glycosylation at the first glycosylation site at residue 181 of PrP, which may involve a non-PrP protein that participates in regulating PrP glycosylation. Because of similar immunoreactivity and enzymatic fragmentation, PrP^Sc^ in VPSPr and fCJD^V180I^ may have an origin similar to iPrP^C^. The possible correlation between human VPSPr and sheep Nor98 is interesting but remains to be further investigated. The low transmissibility of VPSPr and fCJD^V180I^ may result from altered posttranslational modifications including not only glycosylation but also the glycophosphatidylinositol (GPI) anchor. Whether there are any changes in GPI anchor remains unknown. Our current protein sequencing study and glycan analysis of purified rPrP^Sc^ will provide insights into these issues. Future studies with the two diseases and with cell and animal models expressing PrP^V180I^ mutation will help us understand the possible co-factors and molecular mechanisms underlying the formation of the unique prions identified in VPSPr and fCJD^V180I^. 

## References

[1] Prusiner S.B. (1998). Prions. Proc. Natl. Acad. Sci. USA.

[2] Gambetti P., Kong Q., Zou W.Q., Parchi P., Chen S.G. (2003). Sporadic and inherited CJD: Classification and characterisation. Br. Med. Bull..

[3] Giaccone G., Di Fede G., Mangieri M., Limido L., Capobianco R., Suardi S., Grisoli M., Binelli S., Fociani P., Bugiani O., Tagliavini F. (2007). A novel phenotype of sporadic Creutzfeldt-Jakob disease. J. Neurol. Neurosurg. Psychiatry.

[4] Gambetti P., Dong Z., Yuan J., Xiao X., Zheng M., Alshekhlee A., Castellani R., Cohen M., Barria M.A., Gonzalez-Romero D., Belay E.D., Schonberger L.B., Marder K., Harris C., Burke J.R., Montine T., Wisniewski T., Dickson D.W., Soto C., Hulette C.M., Mastrianni J.A., Kong Q., Zou W.Q. (2008). A novel human disease with abnormal prion protein sensitive to protease. Ann. Neurol..

[5] Zou W.Q, Puoti G., Xiao X., Yuan J., Qing L., Cali I., Shimoji M., Langeveld J.P., Castellani R., Notari S., Crain B., Schmidt R.E., Geschwind M., Dearmond S.J., Cairns N.J., Dickson D., Honig L., Torres J.M., Mastrianni J., Capellari S., Giaccone G, Belay E.D., Schonberger L.B., Cohen M., Perry G., Kong Q., Parchi P., Tagliavini F., Gambetti P. (2010). Variably protease-sensitive prionopathy: A new sporadic disease of the prion protein. Ann. Neurol..

[6] Xiao X., Yuan J., Haïk S., Cali I., Zhan Y., Moudjou M., Li B., Laplanche J.L., Laude H., Langeveld J., Gambetti P., Kitamoto T., Kong Q., Brandel J.P., Cobb B.A., Petersen R.B., Zou W.Q. (2013). Glycoform-selective prion formation in sporadic and familial forms of prion disease. PLoS One.

[7] Benestad S.L., Sarradin P., Thu B., Schönheit J., Tranulis M.A., Bratberg B. (2003). Cases of scrapie with unusual features in Norway and designation of a new type, Nor98. Vet Rec..

[8] Pirisinu L., Nonno R., Gambetti P., Agrimi U., Zou W.Q. (2011). Comparative study of sheep Nor98 with human variably protease-sensitive prionopathy and Gerstmann-Sträussler-Scheinker disease. Prion 5..

[9] Zou W.Q., Langeveld J., Xiao X., Chen S., McGeer P.L., Yuan J., Payne M.C., Kang H.E., McGeehan J., Sy M.S., Greenspan N.S., Kaplan D., Wang G.X., Parchi P., Hoover E., Kneale G., Telling G., Surewicz W.K., Kong Q., Guo J.P. (2010). PrP conformational transitions alter species preference of a PrP-specific antibody. J. Biol. Chem..

[10] Zou W.Q., Zheng J., Gray D.M., Gambetti P., Chen S.G. (2004). Antibody to DNA detects scrapie but not normal prion protein. Proc. Natl. Acad. Sci. USA.

[11] Wadsworth J.D., Joiner S., Hill A.F., Campbell T.A., Desbruslais M., Luthert P.J., Collinge J. (2001). Tissue distribution of protease resistant prion protein in variant Creutzfeldt-Jakob disease using a highly sensitive immunoblotting assay. Lancet.

[12] Grasbon-Frodl E., Lorenz H., Mann U., Nitsch R.M., Windl O., Kretzschmar H.A. (2004). Loss of glycosylation associated with the T183A mutation in human prion disease. Acta Neuropathol..

[13] Chasseigneaux S., Haïk S., Laffont-Proust I., De Marco O., Lenne M., Brandel J.P., Hauw J.J., Laplanche J.L., Peoc'h K. (2006). V180I mutation of the prion protein gene associated with atypical PrPSc glycosylation. Neurosci. Lett..

[14] Capellari S., Zaidi S.I., Long A.C., Kwon E.E., Petersen R.B. (2000). The Thr183Ala mutation, not the loss of the first glycosylation site, alters the physical properties of the prion protein. J. Alzheimers Dis..

[15] Zanusso G., Polo A., Farinazzo A., Nonno R., Cardone F., Di Bari M., Ferrari S., Principe S., Gelati M., Fasoli E., Fiorini M., Prelli F., Frangione B., Tridente G., Bentivoglio M., Giorgi A., Schininà M.E., Maras B., Agrimi U., Rizzuto N., Pocchiari M., Monaco S. (2007). Novel prion protein conformation and glycotype in Creutzfeldt-Jakob disease. Arch. Neurol..

[16] Moudjou M., Treguer E., Rezaei H., Sabuncu E., Neuendorf E., Groschup M.H., Grosclaude J., Laude H. (2004). Glycan-controlled epitopes of prion protein include a major determinant of susceptibility to sheep scrapie. J. Virol..

[17] Féraudet C., Morel N., Simon S., Volland H., Frobert Y., Créminon C., Vilette D., Lehmann S., Grassi J. (2005). Screening of 145 anti-PrP monoclonal antibodies for their capacity to inhibit PrPSc replication in infected cells. J. Biol. Chem..

[18] Cong X., Bongarzone S., Giachin G., Rossetti G., Carloni P., Legname G. (2012). Dominant-negative effects in prion diseases: insights from molecular dynamics simulations on mouse prion protein chimeras. J. Biomol. Struct. Dyn..

[19] Salamat M.K., Dron M., Chapuis J., Langevin C., Laude H. (2011). Prion propagation in cells expressing PrP glycosylation mutants. J. Virol..

[20] Tuzi N.L., Cancellotti E., Baybutt H., Blackford L., Bradford B., Plinston C., Coghill A., Hart P., Piccardo P., Barron R.M., Manson J.C. (2008). Host PrP glycosylation: A major factor determining the outcome of prion infection. PLoS Biol..

[21] Nishina K.A., Deleault N.R., Mahal S.P., Baskakov I., Luhrs T., Riek R., Supattapone S. (2006). The stoichiometry of host PrPC glycoforms modulates the efficiency of PrPSc formation *in vitro*. Biochemistry.

[22] Geoghegan J.C., Miller M.B., Kwak A.H., Harris B.T., Supattapone S. (2009). Trans-dominant inhibition of prion propagation *in vitro* is not mediated by an accessory cofactor. PLoS Pathog..

[23] Mutsukura K., Satoh K., Shirabe S., Tomita I., Fukutome T., Morikawa M., Iseki M., Sasaki K., Shiaga Y., Kitamoto T., Eguchi K. (2009). Familial Creutzfeldt-Jakob disease with a V180I mutation: Comparative analysis with pathological findings and diffusion-weighted images. Dement Geriatr. Cogn. Disord..

[24] Jansen C., Head M.W., van Gool W.A., Baas F., Yull H., Ironside J.W., Rozemuller A.J. (2010). The first case of protease-sensitive prionopathy (PSPr) in The Netherlands: A patient with an unusual GSS-like clinical phenotype. J. Neurol. Neurosurg. Psychiatry.

[25] Telling G.C., Scott M., Mastrianni J., Gabizon R., Torchia M., Cohen F.E., DeArmond S.J., Prusiner S.B. (1995). Prion propagation in mice expressing human and chimeric PrP transgenes implicates the interaction of cellular PrP with another protein. Cell.

[26] Supattapone S., Miller M.B., Zou W.Q., Gambetti P. (2013). Cofactor Involvement in Prion Propagation. Prions and Diseases: Physiology and Pathophysiology.

[27] Ma J., Zou W.Q., Gambetti P. (2013). Prion Protein Conversion and Lipids. Prions and Diseases: Physiology and Pathophysiology.

[28] Colby D.W., Prusiner S.B. (2011). Prions. Cold Spring Harb. Perspect. Biol..

[29] Zou W.Q. Transmissible spongiform encephalopathy and beyond (E-letter). Science.

[30] Zou W.Q., Gambetti P. (2004). Modeling of human prions and prion diseases *in vitro* and *in vivo*. Drug Disc. Today: Dis. Mod..

[31] Prusiner S.B. (1982). Novel proteinaceous infectious particles cause scrapie. Science.

[32] Brown P., Gibbs C.J., Rodgers-Johnson P., Asher D.M., Sulima M.P., Bacote A., Goldfarb L.G., Gajdusek D.C. (1994). Human spongiform encephalopathy: The National Institutes of Health series of 300 cases of experimentally transmitted disease. Ann. Neurol..

[33] Tateishi J., Kitamoto T., Hoque M.Z., Furukawa H. (1996). Experimental transmission of Creutzfeldt-Jakob disease and related diseases to rodents. Neurology.

[34] Parchi P., Chen S.G., Brown P., Zou W., Capellari S., Budka H., Hainfellner J., Reyes P.F., Golden G.T., Hauw J.J., Gajdusek D.C., Gambetti P. (1998). Different patterns of truncated prion protein fragments correlate with distinct phenotypes in P102L Gerstmann-Sträussler-Scheinker disease. Proc. Natl. Acad. Sci. USA.

[35] Piccardo P., Manson J.C., King D., Ghetti B., Barron R.M. (2007). Accumulation of prion protein in the brain that is not associated with transmissible disease. Proc. Natl. Acad. Sci. USA.

[36] Zou W.Q., Gambetti P. (2005). From microbes to prions: The final proof of the prion hypothesis. Cell.

[37] Nonno R., Di Bari M., Pirisinu L., D’Agostino C., Marcon S., Riccardi G., Vaccari G., Parchi P., Zou W.Q., Gambetti P., Agrimi U. (2012). Variably protease-sensitive prionopathy is transmissible in bank voles. Prion.

[38] Gambetti P., Xiao X., Yuan J., Cali I., Kong Q., Zou W.Q. (2011). Variably protease-sensitive prionopathy: Transmissibility and PMCA studies. Prion.

[39] Kong Q., Surewicz W.K., Petersen R.B., Zou W.Q., Chen S.G., Parchi P., Capellari S., Goldfarb L., Montagna P., Lugaresi E., Piccardo P., Ghetti B., Gambetti P., Prusiner S.B. (2004). Inherited Prion Diseases. Prion Biology and Diseases.

[40] Yuan J., Dong Z., Guo J.P., McGeehan J., Xiao X., Wang J., Cali I., McGeer P.L., Cashman N.R., Bessen R., Surewicz W.K., Kneale G., Petersen R.B., Gambetti P., Zou W.Q. (2008). Accessibility of a critical prion protein region involved in strain recognition and its implications for the early detection of prions. Cell Mol. Life Sci..

[41] Yuan J., Xiao X., McGeehan J., Dong Z., Cali I., Fujioka H., Kong Q., Kneale G., Gambetti P., Zou W.Q. (2006). Insoluble aggregates and protease-resistant conformers of prion protein in uninfected human brains. J. Biol. Chem..

[42] Zou W.Q., Zou W.Q., Gambetti P. (2013). Insoluble Cellular Prion Protein. Prions and Diseases: Physiology and Pathophysiology.

[43] Zou W.Q., Zhou X., Yuan J., Xiao X. (2011). Insoluble cellular prion protein and its association with prion and Alzheimer diseases. Prion.

[44] Tagliavini F., Prelli F., Ghiso J., Bugiani O., Serban D., Prusiner S.B., Farlow M.R., Ghetti B., Frangione B. (1991). Amyloid protein of Gerstmann-Sträussler-Scheinker disease (Indiana kindred) is an 11 kd fragment of prion protein with an N-terminal glycine at codon 58. EMBO J..

[45] Pirisinu L., Nonno R., Esposito E., Benestad S.L., Gambetti P., Agrimi U., Zou W.Q. (2013). Small ruminant Nor98 prions share biochemical features with human Gerstmann-Sträussler-Scheinker disease and variably protease-sensitive prionopathy. PLoS ONE.

